# Early childhood maltreatment and profiles of resilience among child welfare-involved children

**DOI:** 10.1017/S0954579421001851

**Published:** 2022-02-07

**Authors:** Susan Yoon, Fei Pei, Jessica Logan, Nathan Helsabeck, Sherry Hamby, Natasha Slesnick

**Affiliations:** 1 College of Social Work, The Ohio State University, Columbus, OH, USA,; 2 School of Social Work, Falk College, Syracuse University, Syracuse, NY, USA,; 3 Quantitative Research, Evaluation and Measurement, College of Education and Human Ecology, The Ohio State University, Columbus, OH, USA,; 4 Department of Psychology, The University of the South, Sewanee, TN, USA,; 5 Life Paths Research Center, Sewanee, TN, USA; 6 Department of Human Sciences, College of Education and Human Ecology, The Ohio State University, Columbus, OH, USA

**Keywords:** child maltreatment, early childhood, resilience, latent profile analysis

## Abstract

Given the high burden of child maltreatment, there is an urgent need to know more about resilient functioning among those who have experienced maltreatment. The aims of the study were to: 1) identify distinct profiles of resilience across cognitive, emotional, behavioral, and social domains in young children involved in the child welfare system; and 2) examine maltreatment characteristics and family protective factors in relation to the identified resilience profiles. A secondary analysis was conducted using data from the National Survey of Child and Adolescent Well-Being (NSCAW-II). Latent profile analysis was performed on a sample of 827 children aged 3–5 years (46% girls, Mean age = 3.96). Three distinct resilience profiles were identified: 1) *low cognitive resilience* (24%); 2) *low emotional and behavioral resilience* (20%); and 3) *multidomain resilience* (56%). Caregiver cognitive stimulation, no out-of-home placement, higher caregiver education level, older child age, and being a girl were associated with the *multidomain resilience* profile. The findings provide empirical support for the multifaceted nature of resilience and suggest that practitioners need to help children achieve optimal and balanced development by assessing, identifying, and targeting those domains in which children struggle to obtain competence.

There is a robust body of evidence supporting the long-lasting, negative impact of maltreatment on children’s developmental outcomes. Child maltreatment has been linked to behavior problems, mental health problems, poor academic performance, adolescent substance use and delinquency, and adult criminality (Cicchetti, 2016; Cuadra et al., 2014; Lansford et al., 2010; Lansford et al., 2014; Norman et al., 2012). Although children with maltreatment histories are at significant risk for negative outcomes, some maltreated children exhibit resilience, which is the process of positive adaptation and functioning in the face of adverse life circumstances (Cicchetti, 2013; Dubowitz et al., 2016; Hamby et al., 2018; Meng et al., 2018; Oshri et al., 2017; Yoon et al., 2020). Resilience during early childhood is of particular importance because the basis of core competence is formed during this period, making it a critical window of opportunity for promoting lifelong resilience; yet we know little about how resilience operates in young, maltreated children. Despite theoretical evidence that supports the multidimensionality of resilience, prior empirical research has often employed a simple dichotomous view of resilient versus non-resilient, failing to accurately describe the ways in which resilience outcomes and processes may develop following exposure to child maltreatment. Furthermore, while prior studies have revealed key individual-level predictors (e.g., self-esteem, ego-control, ego-resilience cortisol) of resilience among maltreated children (Cicchetti & Rogosch, 2007; Cicchetti et al., 1993; Flores et al., 2005), less is known about the extent to which family-level protective factors are related to various profiles of resilience in children who have experienced maltreatment. Identifying different profiles of resilience and understanding family ecology as key context for resilience in early childhood is critical to design early interventions that promote optimal resilient functioning across all developmental domains. To address these critical research gaps, we sought to investigate patterns of resilience during early childhood in a child welfare-involved sample of children.

## Child maltreatment and resilience in early childhood

Child maltreatment is a grave public health concern in the United States, with approximately 656,000 victims reported to Child Protective Services (CPS) each year (U.S. DHHS, 2021). It is estimated that 1 in 8 children will experience a confirmed case of maltreatment by 18 years of age (Wildeman et al., 2014). Child maltreatment refers to any acts of commission (e.g., abuse) or omission (e.g., neglect) conducted by a caregiver that results in harm or potential harm to a child (Leeb, 2008). Different types of maltreatment include physical, sexual, emotional abuse, and various forms of neglect, such as physical, emotional, medical, educational neglect, and inadequate supervision. The economic burden of child maltreatment in the United States is substantial, with the estimated average lifetime cost per victim being over $200,000 (Fang et al., 2012). Child maltreatment during the early years of life has a profound negative impact on children’s development across multiple domains – including social, emotional, behavioral, and cognitive functioning (Cicchetti, 2016; Norman et al., 2012). Despite the link between child maltreatment and negative health and developmental outcomes, not all children with a history of child maltreatment experience adverse outcomes. A considerable number of children with maltreatment histories continue to thrive, achieve positive adaptations, and display resilience (Cicchetti, 2013; Cicchetti & Rogosch, 2007; Cicchetti et al., 1993; Dubowitz et al., 2016; Meng et al., 2018; Yoon et al., 2020).

Early childhood – defined in this study as the ages of 3–5 years – is a vital developmental period during which children progress through key developmental processes and establish the basic foundations of competence (Erikson, 1993). Understanding maltreatment and resilience during early childhood is particularly important for several reasons. First, early childhood is a period when children are at the highest risk of experiencing maltreatment (U.S. DHHS, 2021). Ample research suggests that younger children are more vulnerable to child maltreatment. According to the 2019 national child maltreatment data, 45.9% of victims of child maltreatment were between 0 and 5 years of age (U.S. DHHS, 2021). Second, maltreatment experiences in early childhood have significant enduring negative consequences on health and developmental outcomes across the lifespan. For instance, early childhood maltreatment is associated with long-term adverse outcomes, including poor academic performance, altered brain development, psychiatric and emotional problems, unemployment, substance use problems, and chronic diseases (Jedd et al., 2015; Lansford et al., 2014; Norman et al., 2012; Widom, 2014). Finally, early childhood is especially crucial for studying resilience because it represents a critical period for school readiness, with young children actively exploring and developing their socio-emotional, behavioral, and cognitive functioning, which lays the foundation for subsequent successful development (Nelson et al., 2017; Panlilio et al., 2018; Sabol & Pianta, 2012). Unfortunately, we know little about resilience during early childhood among maltreated children because the vast majority of studies have focused on resilience in mid-late childhood and adolescence (Cicchetti & Rogosch, 2012; Collin-Vézina et al., 2011; Martinez-Torteya et al., 2017; Yates et al., 2003), with only a handful of child maltreatment research examining resilience in early childhood (Dubowitz et al., 2016; Sattler & Font, 2018). Improved understanding about resilience during early childhood can offer key information for promoting successful development in the early years of life, which sets the stage for continued success throughout the lifespan.

## Identifying distinct patterns of resilience

To date, there remains a significant amount of confusion and debate in the field on what resilience is and how to best conceptualize this construct (Happer et al., 2017). For example, some scholars define resilience as a personal trait while others define it as a dynamic process (Happer et al., 2017; Luthar et al., 2000). The former approach conceptualizes resilience as an individual’s stable, innate trait that reflects resourceful, sturdy characteristics (Block & Kremen, 1996; Block & Block, 1980). Conversely, the latter conceptualizes resilience as a dynamic developmental process of positive adaptation across multiple domains of functioning following adversity (Cicchetti, 2013; Luthar et al., 2000; Luthar, 2013). Based on empirical evidence providing strong support for resilience as a process (Cicchetti, 2013; Happer et al., 2017; Masten, 1994; Rutter, 1999), we define *resilience* as the outcome or process of positive adaptation in the face of challenging circumstances (Luthar et al., 2000; Masten, 1994).

Drawing from the resilience framework (Garmezy, 1973; Hamby et al., 2018; Masten & Powell, 2003; Yates & Masten, 2004), resilience concerns broad, successful adaptation in multiple domains of functioning – such as cognitive, emotional, behavioral, and social (Dubowitz et al., 2016) – yet resilience is not an “all or nothing” phenomenon (Luthar, 2013). Children may exhibit successful adaptation in some areas (e.g., high cognitive functioning), but not in other areas (e.g., difficulties in social relationships). Many existing studies, however, have either examined only certain domains of resilience (e.g., behavioral resilience) or treated resilience as an overarching construct with little consideration of potential variations in different areas of resilience.

A growing body of research has applied person-centered analytic approaches to examine patterns of resilience among individuals who have experienced child maltreatment. Using latent profile analysis (LPA) and a sample of 12-year-old child victims of alleged maltreatment, one study identified five distinct profiles of adaptation: a) *consistent resilience; b) consistent maladaptation; c) posttraumatic stress problems; d) school maladaptation, family protection; and e) low socialization skills* (Martinez-Torteya et al., 2017). Another LPA study of resilience among maltreated individuals focused on emancipated foster youth and found four distinct profiles of resilience: a) *maladaptive; b) resilient; c) internally resilient; and d) externally resilient* (Yates & Grey, 2012). Using a longitudinal design and a nationally representative sample of maltreated youth, Oshri and colleagues (2018) employed growth mixture modeling and found three classes of resilience/future orientation trajectories: *a) high-persistent; b) low-increasing; and c) high start/decreasing*. A recent study focused on patterns of resilience/competence across multiple domains of functioning in a high-risk sample of emerging adults and found four distinct patterns of functioning: *a) multifaceted competence; b) multiproblem; c) externalizing problems; and d) work/school impairment* (Russotti et al., 2020). Collectively, these studies suggest emerging empirical evidence for heterogeneous patterns of adaptation and resilience following child maltreatment. Despite the growing application of person-centered approaches to resilience in maltreatment samples, the bulk of previous work has focused on older children and adolescents, and these approaches have not been widely applied to younger children with maltreatment experiences. Discovering distinct profiles of resilience during early childhood is an important new question to move the field forward and inform the development of early interventions that can promote optimal and balanced functioning across different domains of development in young children with maltreatment experiences.

## Maltreatment characteristics and family ecology as critical contexts for resilience

Child maltreatment is a complex phenomenon, which may not be accurately represented by a simple dichotomous status (Manly, 2005). However, much of prior work in resilience has examined maltreatment as an overarching, dichotomous construct (i.e., maltreatment vs. non-maltreatment) or focused only on a single type of maltreatment (e.g., sexual abuse). Maltreatment characteristics, such as the type and nature of maltreatment and out-of-home placement, can play important roles in understanding childhood resilience. McLaughlin and Sheridan (2016) stress the need for adopting a dimensional approach to childhood adversity, moving beyond the cumulative risk approach, to account for unique nature and core underlying dimensions (i.e., *threat* and *deprivation*) of adversity. Drawing from this approach, examining different dimensions of child maltreatment, such as child neglect (i.e., omission/deprivation) and child abuse (i.e., commission/threat; McLaughlin & Sheridan, 2016), in relation to childhood resilience might be useful. In studies examining behavioral outcomes, different dimensions/forms of maltreatment have been associated with various levels and patterns of behavioral adjustment. Child neglect (i.e., high deprivation) tended to be related to greater internalizing symptoms (Bolger & Patterson, 2001, Manly et al., 2001) whereas physical and emotional abuse (i.e., high threat) were found to be more related to externalizing symptoms (Manly et al., 2001; Teisl & Cicchetti, 2008; Villodas et al., 2016). Out-of-home placement, including entry into foster care, has also been associated with higher rates of behavior problems (Berger et al., 2009; Proctor et al., 2010). Similar effects may be found for the development of various profiles of resilience among young, maltreated children.

The wider family and caregiving environment also plays an important role in the development of childhood resilience in the context of child maltreatment. Families at risk of maltreatment may have unique challenges and strengths that may inhibit or promote the manifestation and dynamic change in resilience. Poverty, lower socioeconomic status (SES), and parental psychopathology are prevalent among families of maltreated children (Conrad-Hiebner & Byram, 2020; Doidge et al., 2017; Garcia et al., 2017) and have been found to hamper resilient development of children (Dubowitz et al., 2016; Jaffee & Gallop, 2007). Conversely, the presence of protective factors in the home can foster resilience in maltreated children. Higher levels of parental emotional support, cognitive stimulation, and caregiver stability have been associated with positive developmental outcomes in children who have experienced child maltreatment (Bell et al., 2013; Proctor, et al., 2010; Sattler & Font, 2018; Yoon, 2018).

## The current study

This study has several strengths and contributions, including its focus on early childhood resilience, application of a person-centered analytic approach (i.e., LPA) to examine resilience profiles, and utilization of the National Survey of Child and Adolescent Well-Being (NSCAW-II) dataset that contains a nationally representative sample of child welfare-involved children and well-validated, developmentally appropriate measures for clinical assessments of children. The first aim of the current study was to identify profiles of resilience across multiple domains of functioning (cognitive, emotional, behavioral, social) during early childhood among child welfare-involved children. Building upon prior research that found three to five distinct classes of resilience/competence in maltreatment samples (Martinez-Torteya et al., 2017; Oshri et al., 2018; Russotti et al., 2020), we hypothesized that we would identify at least four distinct profiles of resilience (e.g., multidomain resilience, multidomain problems, low behavioral resilience, and low cognitive resilience). The second aim was to investigate maltreatment characteristics and family protective factors as predictors of resilience profiles. We hypothesized that exposure to both abuse and neglect would be associated with poorer profiles of resilience (e.g., low competence in all domains) while better quality of caregiving/family environment (e.g., higher parental educational level, greater parental emotional support and cognitive stimulation, remaining in the home after CPS investigation) would be associated with better profiles of resilience (e.g., high competence in all domains).

## Methods

### Participants and procedures

A secondary data analysis was conducted using the NSCAW-II data (Dowd et al., 2013) which includes a national probability sample of children involved with the child welfare system in the United States. Utilizing a two-stage stratified sample design, 5872 children (ages 0–17.5 years) who had contact with the child welfare system at the time of sampling (February 2008–May 2009) were recruited from 81 Primary Sampling Units in 30 US states. Data were collected across three waves (W1: baseline, W2: 1.5-year follow-up, W3: 3-year follow-up) from 2008 to 2012. The present study utilizes data collected at Wave 1, and the study sample consists of 827 children who were 3–5 years of age at W1.

Table 1 summarizes sample characteristics. Children’s ages ranged from 3 to 5 years, with a mean age of 3.96 (*SD* = .82). About half of the sample was girls (46.1%) and 39.6% was White non-Hispanic, 31.4%, Black non-Hispanic, 24% Hispanic, and 5% other race (American Indian, Asian, Native Hawaiian/Pacific Islander, multiple race). Caregivers’ ages ranged from 18 to 74 years (mean age = 34.04, *SD* = 11.42). About 46.1% of the caregivers identified as White non-Hispanic, 27.4% as Black non-Hispanic, 20.6% as Hispanic, and 5.4% as other race (American Indian, Asian, Native Hawaiian/Pacific Islander, multiple race). Approximately 72% of the children lived in the home and the remaining 28% lived in out-of-home care (e.g., foster care, kinship care, group homes, and residential programs). The mean duration of time with the current caregiver was 37 months. About half (49%) of the caregivers were employed (either full-time or part-time) and 74.2% completed high school or more education. About 78.1% of the caregivers reported household income at or below the 200% federal poverty level.

### Measures

#### Resilience

Resilience was assessed using multiple instruments. For the cognitive domain, verbal ability (expressive language skills) and receptive language skills were measured using the *Preschool Language Scale expressive communication sub-scale and auditory comprehension sub-scale, respectively (PLS-3*; Zimmerman et al., 1992). The PLS-3 is a standardized tool of overall language development of children ages 0–6 years that has demonstrated strong validity as evidenced by its significant correlation with the Peabody Picture Vocabulary Test-III (PPVT-III; Dunn & Dunn, 1997) (Qi & Marley, 2011). Internal consistencies of the scales were good in this sample (expressive communication scale: *α* = .87, auditory comprehension scale: *α* = .85). For the social domain, the *Social Skills Rating System (SSRS; 39 items;* Gresham & Elliott, 1990) was used to measure caregiver perceptions of children’s prosocial behavior (e.g., self-control, assertion, responsibility, cooperation) and the *Vineland Adaptive Behavior Scale Screener (VABSS; 15 items*; Sparrow et al., 1993) was used to assess caregiver report of child functioning in social situations (e.g., play, interpersonal relations). Internal consistencies were good in this sample (SSRS: *α* = .91, VABSS: *α* = .75). For the emotional domain, emotion regulation and anxiety/depression were assessed using the *Child Behavior Checklist (CBCL/1.5–5*; Achenbach & Ruffle, 2000) emotionally reactive scale (8 items; *α* = .78) and anxious/depressed scale (8 items; *α* = .63), respectively. For the behavioral domain, child aggressive behavior and attention were measured using the CBCL aggression scale (8 items; *α* = .82) and attention problems scale (8 items; *α* = .91). The CBCL has demonstrated strong reliability and validity, including convergent and discriminative validity, with the measure showing significant associations with the DSM-oriented scales (Nakamura et al., 2009). For caregiver reports of child functioning (i.e., CBCL, SSRS, VABSS), the child’s current caregiver completed the measures. When the biological parent was the current caregiver, the child had been in the parent’s care on average for about 45 months (*M* = 44.83, *SD* = 13.82). When the child was in out-of-home care and a non-biological parent was the current caregiver (e.g., foster care parent, kinship caregiver), the child had been in their care on average for a little over a year (*M* = 13.57, *SD* = 17.71). Descriptive statistics and bivariate correlations for resilience indicators are presented in Table 2.

#### Maltreatment characteristics

Three maltreatment-related constructs were assessed: child abuse, child neglect, and placement status. The information about the type of maltreatment that led to the referral to CPS was obtained from caseworkers who were asked to identify the type(s) of alleged maltreatment, using the *Modified Maltreatment Classification System (MMCS;* English & the LONSCAN Investigators, 1997). Based on the dimensional approach to childhood adversity (McLaughlin & Sheridan, 2016), two different forms/dimensions of maltreatment were considered: child abuse (i.e., commission/high threat) and child neglect (i.e., omission/high deprivation).

*Child abuse* was assessed using the caseworkers’ reports on physical abuse, emotional abuse, and sexual abuse. Caseworkers responded yes or no to the three non-mutually exclusive abuse type items to indicate whether the type(s) of abuse was reported to CPS. The child was considered to have experienced child abuse (= 1) if the caseworker endorsed any of the three items. *Child neglect* was assessed using two caseworker-reported child neglect items: physical neglect and supervisory neglect (failure to provide supervision). The child was considered to have experienced child neglect ( = 1) if the caseworker endorsed either or both of the two items. The *placement status (out-of-home placement)* was assessed using the caseworkers’ reports about the placement of children in out-of-home care (0 = *no*, 1 = *yes*).

#### Caregiver protective factors

*Caregiver education* (0 = *less than high school*, 1 = *high school or more*) was measured using caregiver’s self-report. *Cognitive Stimulation* and *emotional support* were measured using the Early Childhood Home Observation for Measurement of the Environment (EC-HOME; Caldwell & Bradley, 1984). The EC-HOME, designed to be used for children ages between 3 and 6 years, contains items that assess the physical home environment and the caregiver’s behaviors toward the child. The EC-HOME obtains data using interviews with caregivers and through observations by the interviewers. The cognitive stimulation scale (14 items; e.g., read stories to the child; takes the child to the museum; # of books child has of his/her own) and the emotional support scale (12 items; caregiver responds verbally to child’s speech; caregiver caressed, kissed, or hugged child) assessed caregivers’ cognitive responsiveness and emotional support, respectively.

#### Control variables

Child age, sex, and race/ethnicity were reported by caregivers. For race/ethnicity, the following dummy variables were used: White non-Hispanic, Hispanic, and Other (American Indian, Asian, Native Hawaiian/Pacific Islander, multiple race). Black non-Hispanic was used as a reference group. Household income (0 = *over 200% federal poverty level*; 1 = *at or below 200% federal poverty level*) was measured using caregiver self-report.

### Data analysis

To identify profiles of resilience among young children involved with the child welfare system, we conducted latent profile analysis (LPA), which is a type of latent class analysis that is used to empirically classify individuals into groups based on their responses on several continuous indicators (Collins & Lanza, 2009). LPA is a person-centered analysis that assumes that heterogeneity in the variance and covariance of the indicator variables is caused by an underlying grouping variable and seeks to place respondents into groups accordingly. Essentially, the LPA explores whether latent profiles exist within the observed data and provides a framework for identifying how many profiles exist. With that information, we could characterize the identified profiles and determine which persons belong to which group. LPA uses the scores of each person on each observed variable as well as the co-variation between observed variables to determine possible group membership for each person included in the data.

We used the three-step approach LPA (Asparouhov & Muthén, 2018) to address the research aims. Specifically, we performed the manual maximum likelihood (ML) three-step approach that has been recommended as one of the best practices in including auxiliary variables in mixture models (Nylund-Gibson et al., 2019). In the first step, the best-fitting unconditional model was identified. Because it was unclear from the extant literature how many profiles we might expect to find in resilient functioning based on our included manifest variables, we fitted a series of LPA models, with 2–10 groups identified. We used guidelines set forth by Nylund et al., (2007) to determine the best fitting model. We compared the model fits using the Bayesian information criterion (BIC), the Lo-Mendell-Rubin likelihood ratio test (LMR), and the bootstrap likelihood ratio test (BLRT). The model with the lowest BIC and significant LMR and BLRT test was considered the potential best fitting model. Further consideration was given to the separation and uniqueness of the profile identifications: a high entropy value (greater than .8, or closest to 1.0) (Ramaswamy et al., 1993), and no less than 5% of the total count in any class (Hipp & Bauer, 2006). In addition to these criteria, the features of the identified profiles were examined to determine whether they align with current theoretical understandings of children’s development (Logan & Pentimonti, 2016). The final decision on which model to select was a balance of all indicators described above. The second step of the three-step approach involved computing the estimated conditional probabilities for modal class assignment (Asparouhov & Muthén, 2018; Nylund-Gibson et al., 2019). The final third step involved fitting the auxiliary model with covariates. In this step, the most likely class variable (modal class assignment) was used as a nominal latent class indicator (‘*n*’) with uncertainty rates (i.e., classification error) fixed at probabilities obtained from the second step. The focal predictors and control variables were added as covariates to the conditional LPA model whereby class membership was regressed on covariates. In terms of the amount of missing data on the indicators of resilience, receptive language skills had the largest missing proportion (20.2%), followed by verbal ability (20.7%) and prosocial skills (3.7%). All other indicators had no missing data. Missing data were handled using the full information maximum likelihood (FIML) methods (Enders & Bandalos, 2001). LPA analyses were conducted using Mplus v.8 (Muthén & Muthén, 1998–2017) and descriptive statistics were performed using SPSS v. 27.

## Results

### Resilience profiles

Table 3 displays model fit indices for the LPA models of resilience profiles (fit indices are not presented beyond the 5-class solution because they did not provide meaningful or helpful information for model selection). The model fit indices yielded mixed information regarding the optimal number of classes. The 10-class model had the lowest BIC score, but 2-class to 5-class models each had a significant LMR value. BLRT statistics were significant in all models, adding no meaningful information. Due to the lack of consensus or clear indication regarding the optimal number of classes based on the model fit indices, we gave further consideration into the quality of the classification, model parsimony, size of each class, and conceptual meaningfulness and interpretability of the classes. Specifically, the 3-class and 4-class solutions showed equally good fit indices (e.g., low BIC, significant LMR and BLRT statistics), but the examination of graphic outputs and interpretability of the classes confirmed that the 3-class model had the most distinct and meaningful classes. The 4-class solution included two profiles that did not have conceptually meaningful differences, with both profiles representing children (similarly) faring well across multi-domain of resilience. For parsimony, we selected the 3-class solution as the final model. Additionally, the 3-class model revealed a relatively high-quality classification with an entropy of .85 and adequate sample size for each of the 3 identified classes.

**Figure 1.**
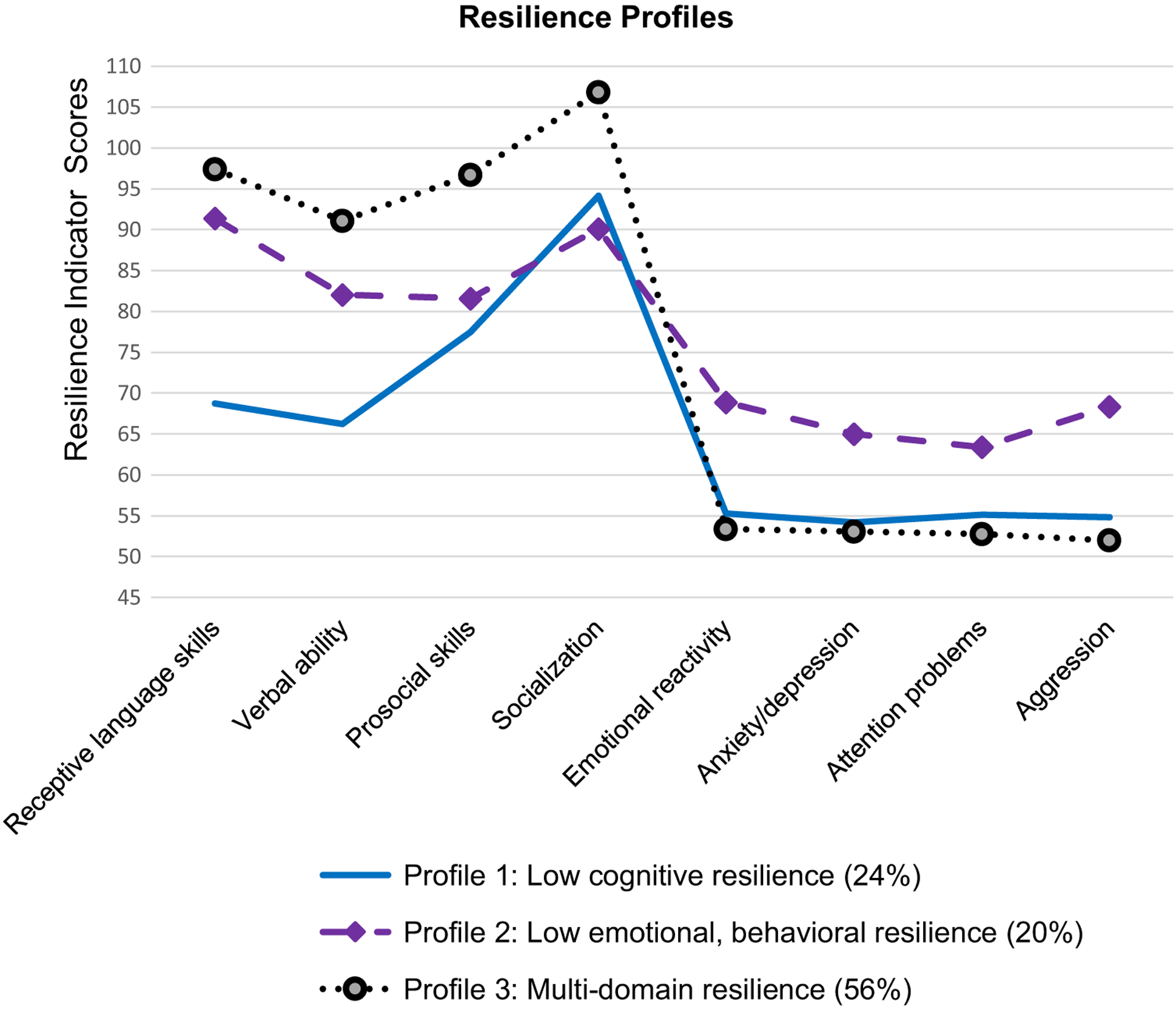
Latent profiles of early childhood resilience. For receptive language skills, verbal ability, prosocial skills, and socialization, scores between 85 and 115 are considered to be within the normal range. For the emotional reactivity, anxiety/depression, attention problems, and aggression scales, scores <65 are considered to be within the normal range, scores from 65 to 69 are considered to be borderline, and scores >69 are considered to be clinically significant.

The 3-class resilience profile model (Figure 1) contained the following profiles: 1) *low cognitive resilience* (24%); 2) *low emotional and behavioral resilience* (20%); and 3) *multi-domain resilience* (56%). The *low cognitive resilience* profile (24%) included children who showed below the normal level (scores < 85) of expressive and auditory language development, moderate level of social functioning, and positive emotional and behavioral functioning (i.e., emotional and behavior problems scores within the normal range). The *low emotional and behavioral resilience* profile (20%) consisted of children whose emotional and behavioral problems scores were in the borderline range. Children with this profile had moderate levels of cognitive and social functioning. The *multi-domain resilience* profile (56%) included children who exhibited positive adaptation (within the normal range) and competence across all domains of functioning. Table 4 shows mean scores on resilience indicators for each class.

### Predictors of resilience profiles

A shown in Table 5, children placed in out-of-home care were more likely to be in the low emotional and behavioral resilience group compared to the *multi-domain resilience* group (OR = 1.70, CI = 1.01–2.85, *p* = .046) and the *low cognitive resilience* group. For children who had caregivers with high school or more education, the likelihood of being in the low cognitive resilience group decreased by 72% compared to the *multi-domain resilience* group (OR = .28, CI = .12–.65, *p* = .003) and by 82% compared to the *low emotional and behavioral resilience* group (OR = .18, CI = .07–.47, *p* < .001). For every point higher in caregiver cognitive stimulation, the odds of being in the *low cognitive resilience* group and the low emotional and behavioral resilience group compared to the *multi-domain resilience* group decreased by 34% (OR = .66, CI = .55–.80, *p* < .001) and 18% (OR = .82, CI = .73–.92, *p* = .001), respectively. Additionally, for every point higher in caregiver cognitive stimulation, the odds of being in *low cognitive resilience* group compared to the *low* emotional and behavioral resilience group decreased by 20% (OR = .80, CI = .67–.97, *p* = .023). Girls had 55% lower odds of being in the *low cognitive resilience* group (OR = .45, CI = .24–.87, *p* = .017) and the low emotional and behavioral resilience group (OR = .45, CI = .28–.71, *p* = .001), compared to the *multi-domain resilience* group. For every one year increase in children’s age, the odds of being in the *low cognitive resilience* group (OR = .05, CI = .02–.12, *p* < .001) and the low emotional and behavioral resilience group (OR = .70, CI = .50–.97, *p* = .031) compared to the *multi-domain resilience* group decreased by 95% and 30%, respectively. Further, for every one year increase in age, the odds of being in *low cognitive resilience* group compared to the *low* emotional and behavioral resilience group decreased by 92% (OR = .08, CI = .03–.19, *p* < .001).

## Discussion

The current study sought to identify distinct profiles of early childhood resilience across the cognitive, emotional, behavioral, social domains among child welfare-involved children. This study makes important contributions to the field in two ways. First, this study examines resilience within an understudied developmental stage – early childhood. Resilience during early childhood is important because it lays the foundation for continued, life-long resilience, yet little is known about early childhood resilience in the context of child maltreatment (Yoon et al., 2019). Second, this study focuses on unraveling distinct patterns of early childhood resilience through a novel application of a person-centered analytic approach (LPA) in examining resilience, going beyond the traditional variable-centered analytic approach. Although person-centered approaches are not new, this approach has not yet been rigorously applied to understand heterogeneity in resilience during early childhood.

Consistent with prior studies that used person-centered approaches to examine resilience following child maltreatment, we found heterogeneous and distinct profiles of resilience during early childhood among child welfare-involved children. Specifically, we identified three unique profiles of resilience functioning: *low cognitive resilience* (24%), *low emotional and behavioral resilience* (20%), and *multi-domain resilience* (56%). To our knowledge, this is the first study to delineate various profiles of resilience using a national probability sample of children in the child welfare system. Our findings corroborate past person-centered studies that found different patterns of resilience among individuals with a history of maltreatment (Martinez-Torteya et al., 2017; Russotti et al., 2020; Yates & Grey, 2012) and offer compelling evidence for the multifaceted nature of resilience.

It is worth noting that over half of the sample (57%) was classified into the *multi-domain resilience* group where children displayed resilient functioning in all assessed domains. This finding is encouraging, considering that we used a high-risk sample of children, and that the assessment of resilience was conducted soon after their involvement with the child welfare system. The finding that the *multi-domain resilience* group contained the largest portion of the sample is also consistent with prior resilience profiles studies that found a profile of multi-dimensional resilience to be most common among high-risk samples of children exposed to family violence (e.g., McDonald et al., 2016: 66%; Yates & Grey, 2012: 47%). Further, such finding is generally in line with prior (variable-centered) studies that estimated approximately half of children to show resilience/competence across multiple domains of functioning following exposure to child maltreatment (Dubowitz et al., 2016, Yoon et al., 2020). Together, these findings suggest that children with exposure to trauma and adversities are not predetermined to maladaptation and failure. On the flip side, however, the findings suggest that approximately half of children may be struggling in achieving adaptations in at least some areas of development and functioning. Further, given that the measurement of resilience was performed at one time point representing just a snapshot of resilience, more research is needed to understand the progression and changes in resilience patterns over time.

The other two profiles (*low cognitive resilience* [24%], *low emotional and behavioral resilience* [20%]) together represented a little less than half of the sample. The *low cognitive resilience* profile and the *low emotional and behavioral resilience* profile validate the notion that resilience is not an “all or nothing” phenomenon (Luthar, 2013) in that children with these profiles displayed more resilience in certain areas while showing less resilience in other areas. The *low cognitive resilience* profile appears to align with school maladaptation profiles identified in prior research (e.g., “school maladaptation/family protection profile” in Martinez-Torteya et al., 2017; “work/school impairment” profile in Russotti et al., 2020). However, the *low cognitive resilience* profile identified in the current study is unique in that it was based on specific measures of language/cognitive development and pinpoint a group with less resilience in language development, whereas the school maladaptation profiles in previous studies encompassed broader indicators of school adaptation (e.g., educational attainment, employment status, peer relations) beyond language/cognitive ability. Low level of language development featured in this profile is consistent with prior research that found the harmful effects of child maltreatment, particularly child neglect, on language competence (Culp et al., 1991; Lum et al., 2018). Yet, considering that this study focused on young children in early childhood, it is possible that children with this profile develop increased competence in language/cognitive functioning over time as they age.

The *low emotional and behavioral resilience* profile was characterized by children’s emotional and behavioral problems scores in the borderline range, with emotional reactivity and aggression scores approaching clinical significance. This profiles is similar to emotional and behavioral maladaptation profiles identified in prior studies (e.g., “externalizing problems” profile in Russotti et al., 2020) and also aligns with a robust body of evidence suggesting the link between child maltreatment and psycho-behavioral problems (Jaffee, 2017, Yoon et al., 2017). However, given that externalizing behaviors, especially aggressive behavior, tend to peak during early childhood and gradually decrease over time (Gilliom & Shaw, 2004), higher levels of emotional and behavioral symptoms in this profile may merely reflect normative developmental patterns and naturally decline with age.

In terms of potential predictors of resilience profiles, we found no relationships between maltreatment types and resilience profiles. Neither child abuse nor child neglect significantly predicted membership in resilience profiles. The null findings in the current study may be partially explained by the use of simple binary maltreatment variables in the analysis. We used two dichotomous (yes vs no) variables to assess exposure to child abuse and exposure to child neglect, yet these two variables are likely highly correlated with each other given that co-occurrence of maltreatment subtypes is common (Kim et al., 2017; Warmingham et al., 2019). Considering that all children in our sample had been involved with the child welfare system due to alleged child abuse and neglect and that multiple maltreatment types, including abuse and neglect, often co-occur (Vachon et al., 2015; Warmingham et al., 2019), more nuanced and informative measures of maltreatment that fully capture the frequency, severity, co-occurrence and chronicity of maltreatment may have been useful in discriminating maltreatment characteristics across different profiles of resilience.

As expected, out-of-home placement was associated with the *low emotional and behavioral resilience* profile. This finding corroborates prior studies that found higher levels of emotional and behavioral problems, such as internalizing and externalizing symptoms, among children in out-of-home care (e.g., foster care) compared to those in the home (Oswald et al., 2010; Turney & Wildeman, 2016). It is worth noting that a bidirectional relationship may exist between out-of-home placement and the *low emotional and behavioral resilience* profile. Out-of-home placement can signal more severe maltreatment and children in out-of-home care may already have had higher levels of behavior problems resulting from severe maltreatment, but out-of-home placement can also contribute to the development or exacerbation of emotional and behavior problems given that a separation from parents is a highly traumatic experience for children (Melinder et al., 2013).

With regard to caregiver/family protective factors, caregiver education was found to be a salient predictor of membership in the *low cognitive resilience* group. Children who had caregivers with high school or more education were less likely to be in the *low cognitive resilience* group compared to children of caregivers with less than high school education. This finding is similar to previous research findings that reported a positive association between parental educational level and child literacy/achievement (Davis-Kean, 2005; Goltermann et al., 2020). Prior research has suggested the roles of both genetic (e.g., parents’ IQ) and environmental (e.g., cognitive stimulation) factors in the intergenerational transmission of cognitive abilities (Tucker-Drob & Harden, 2012). Caregivers who have higher education levels may have greater resources to provide their children with the knowledge and skills they need to excel in cognitive development.

Relatedly, our results revealed that greater cognitive stimulation from caregivers predicted a lower likelihood of children being in the *low cognitive resilience* group and the *low emotional and behavioral resilience* group compared to the *multi-domain resilience* group. These findings are consistent with prior literature that supports the positive impact of parental investments and cognitive stimulation (e.g., singing song and reading books to children, teaching letters and numbers, or visiting museums) on children’s cognitive development during early childhood (Cabrera et al., 2020; Harris et al., 2014; Hubbs-Tait et al., 2002; Tucker-Drob & Harden, 2012). Collectively, these findings indicate that broader family and environmental factors – beyond specific maltreatment episodes and experiences – may be important in determining different profiles of resilience among child welfare-involved children.

Finally, children’s demographic characteristics, such as age and sex, were predictive of resilience profiles. Specifically, older children were less likely to be in the *low cognitive resilience* group compared to the *multi-domain resilience* group or the *low emotional and behavioral resilience* group, suggesting that child age was closely related to the *low cognitive resilience* profile. Considering that cognitive resilience was defined by performance on vocabulary measures, this finding is consistent with the rapid developmental trajectory of vocabulary in early childhood (typical gain from age 3 to age 4 is approximately one full standard deviation, Schmitt et al., 2017). It would be important to monitor the progress of language development of children in the *low cognitive resilience* group and examine if they continue to struggle in cognitive development or if they display improving cognitive functioning as they grow older.

Another interesting finding was that girls, compared to boys, were more likely to show the *multi-domain resilience* profile. This result is consistent with studies that found girls and women to display greater resilience following maltreatment (Davidson-Arad & Navaro-Bitton, 2015; Fava et al., 2018; McGloin & Widom, 2001; Oshri et al., 2018). Our findings most closely align with McGloin and Widom’s (2001) work that found women showing resilience across a wider array of domains of functioning, including social activity, high school graduation, successful employment, no psychiatric disorder, no substance use, than men. These findings seem to suggest that girls and women are generally more resilient than boys and men. Importantly, however, Oshri et al. (2018) found that despite the overrepresentation of girls in a positive pattern of future orientation – a key component of resilience – a fraction of girls diverged from this tendency and showed elevated maladjustment and less positive outcomes than boys. Taken together, no conclusions can be drawn regarding sex and resilience, and further research is necessary to understand the extent to which and under what conditions girls show greater resilience after experiencing maltreatment.

### Limitations

Our study findings need to be considered in light of several limitations. First, we used cross-sectional data which limits our ability to make any causal inferences among study variables. Relatedly, we were unable to examine the change in resilience profiles over time due to the cross-sectional nature of the study design. Second, our study sample consisted of children who have been involved with the child welfare system due to alleged child abuse and neglect. Without a comparison group of children who have not been involved with the child welfare system, it is challenging to interpret the true meaning of the latent profiles identified in this study. For example, it is difficult to know if children in the *multi-domain resilience* group exemplified less maladaptation, normal level of functioning, or high competence compared to non-child welfare-involved children. Further, the lack of a comparison group in the study limits the generalizability of the study results to broader populations. Third, the use of psychopathology measures (i.e., the CBCL) in measuring emotional and behavioral resilience is a limitation. Although we acknowledge that there is an ongoing debate about the use of the measures of psychopathology when assessing resilience (Walsh et al., 2010), we were limited to the data already collected in the NSCAW-II, and unfortunately there were no measures of positive functioning (as opposed to measures of problematic functioning and psychopathology) available for the emotional and behavioral domains. Finally, it should be noted that the length of time with the current caregiver ranged from 1 month to 72 months, suggesting that the duration of the observation period on which the current caregiver based to rate the child’s socio-emotional and behavioral functioning may have varied. For instance, some caregivers had had a child in their care only for a month at the time of survey and relied on 1 month of observation to report on the child even though the time frame for item responses was the past 6 months for some measures. As such, the varying duration of care, especially when the length was less than 6 months, may have threatened the accuracy and validity of the caregivers’ ratings.

### Implications

The findings of the current study offer important implications for practice and research.

This study demonstrates the potential usefulness and applicability of a person-centered analytic approach (i.e., latent profile analysis) in examining heterogeneity in resilience. Additionally, the findings of distinct and different profiles of resilience across multiple domains of functioning highlight the importance of treating resilience as a multifaceted construct as well as considering specific, individual dimensions of resilience when studying resilience. In light of the limitations of the current study, future studies should examine changes in resilience profiles over time, using longitudinal data. Furthermore, future research should consider testing the profile solution against proximal and distal outcomes to understand and confirm the validity of the identified profiles.

The distinct profiles of resilience that we identified may prove useful for practitioners working with child welfare-involved children. Specifically, we find that children may exhibit positive adaptation in some areas (e.g., high cognitive functioning) but not in other areas (e.g., difficulties in social relationships). Our work suggests that practitioners should intentionally consider the heterogeneous nature of resilience when working with children who have experienced early childhood maltreatment. Further, rather than focusing only on reducing psychopathology, practitioners could instead consider adopting strengths-based approaches; assessing and identifying areas of strength for children, while also targeting those domains in which children struggle to obtain competence and help them achieve optimal and balanced development. Future work developing and testing resilience-promoting interventions should also consider factors that may be associated with certain resilience profiles or patterns. For example, the present study demonstrated that the percentage of children in out-of-home care was significantly higher in the low emotional, behavioral resilience compared to that of the other two resilience classes. Therefore, one could test whether interventions that facilitate positive emotional and behavioral functioning may be particularly important for children in out-of-home placement. Similarly, based on our findings that highlight caregiver cognitive stimulation as a key promotive factor for cognitive resilience, parenting support for creating a learning-rich home environment could be incorporated into interventions that aim to promote cognitive resilience among young children with maltreatment histories.

## Figures and Tables

**Table 1.** Sample characteristics (*N* = 827)

	%	*M (SD)*	Range
Child characteristics			
Age (in years)		3.96 (0.82)	3–5
Sex (girls)	46.1%		
Race/ethnicity			
White; Non-Hispanic	39.6%		
Black; Non-Hispanic	31.4%		
Hispanic	24.0%		
Other	5.0%		
Caregiver characteristics			
Age (in years)		34.04 (11.42)	18–74
Race/ethnicity			
White; Non-Hispanic	46.1%		
Black; Non-Hispanic	27.4%		
Hispanic	20.6%		
Other	5.4%		
Setting			
In-home	71.8%		
Formal kin care	7.9%		
Informal kin care	7.2%		
Foster care	12.4%		
Group homes, residential programs, and other	0.5%		
Caregiver (blood) relationship to the child			
Biological mother	62.8%		
Biological father	7.0%		
Grandparents	9.7%		
Other relatives	5.7%		
No blood relationship	14.9%		
Duration of care (in months)		37.32 (20.33)	1–72
Caregiver employment (employed)	49.0%		
Caregiver’s education (less than high school)	25.8%		
Household income ≤200% poverty level	78.1%		

*Note*. Other race included American Indian, Asian, Native Hawaiian/Pacific Islander, and Multiple race categories.

**Table 2.** Correlations among indicators of resilience

	*M (SD)*	1	2	3	4	5	6	7
1. Receptive language skills standard score	89.50 (19.89)	-						
2. Verbal ability standard score	83.52 (20.48)	.64**	-					
3. Prosocial skills standard score	89.27 (16.62)	.28**	.28**	-				
4. Socialization standard score	100.54 (19.02)	.22**	.28**	.59**	-			
5. Emotional reactivity T score	56.88 (8.35)	−.03	−.03	−.23**	−.22**	-		
6. Anxiety/depression T score	55.65 (7.05)	.01	−.02	−.18**	−.20**	.70**	-	
7. Attention problems T score	55.40 (6.32)	−.04	−.09*	−.29**	−.31**	.54**	.49**	-
8. Aggression T score	55.85 (8.90)	−.05	−.08	−.36**	−.30**	.71**	.54**	.62**

* *p* < .05.

** *p* < .01.

**Table 3.** Model fit indices for resilience LPA models

Model	BIC	LMR	BLRT	Profile size	Entropy
2-class	16571.07	1309.67***	−8867.23***	Profile 1: 27.0%, Profile 2: 73.0%	.87
**3-class**	**15837.82**	**780.80** ***	−8201.56***	**Profile 1: 24.5%, Profile 2: 19.6%, Profile 3: 55.9%**	**.85**
4-class	15534.89	357.48***	−7804.71***	Profile 1: 21.7%, Profile 2: 43.8%, Profile 3: 25.7%, Profile 4: 8.8%	.84
5-class	15367.29	224.34*	−7623.01***	Profile 1: 7.8%, Profile 2: 27.0%, Profile 3: 16.4%, Profile 4: 9.6%, Profile 5: 39.2%	.84

* *p* < .05.

*** *p* < .001.

**Table 4.** Means of resilience indicators in the 3-class model

		Resilience Profiles				
	Total sample	Profile 1 (24%): low cognitive resilience	Profile 2 (20%): low emotional, behavioral resilience	Profile 3 (56%): multidomain high resilience		
Indicators	*M (SD)*	*M (SD)*	*M (SD)*	*M (SD)*	*F*	Post hoc comparisons
Receptive language skills standard score	89.50 (19.29)	68.72 (15.30)	91.36 (18.16)	97.40 (15.66)	173.98***	1 ≠ 2,3; 2 ≠ 3
Verbal ability standard score	83.52 (20.48)	66.19 (13.85)	82.05 (18.07)	91.07 (19.14)	104.99***	1 ≠ 2,3; 2 ≠ 3
Prosocial skills standard score	89.27 (16.62)	77.51 (15.72)	81.58 (13.54)	96.75 (13.72)	149.97***	1 ≠ 2,3; 2 ≠ 3
Socialization standard score	100.54 (19.02)	94.16 (19.11)	90.09 (16.77)	106.82 (17.18)	71.49***	1 ≠ 3; 2 ≠ 3
Emotional reactivity T score	56.88 (8.35)	55.26 (5.89)	68.90 (8.00)	53.40 (4.82)	436.50***	1 ≠ 2,3; 2 ≠ 3
Anxiety/depression T score	55.65 (7.05)	54.18 (5.22)	64.98 (8.09)	53.05 (4.02)	311.58***	1 ≠ 2,3; 2 ≠ 3
Attention problems T score	55.40 (6.32)	55.12 (5.26)	63.38 (6.67)	52.77 (3.81)	289.35***	1 ≠ 2,3; 2 ≠ 3
Aggression T score	55.85 (8.90)	54.83 (5.88)	68.35 (11.36)	55.85 (8.90)	409.70***	1 ≠ 2,3; 2 ≠ 3

*Note*. For receptive language skills, verbal ability, prosocial skills, and socialization, scores between 85 and 115 are considered to be within the normal range. For the emotional reactivity, anxiety/depression, attention problems, and aggression scales, scores <65 are considered to be within the normal range, scores from 65 to 69 are considered to be borderline, and scores >69 are considered to be clinically significant.

*** *p* < .001.

**Table 5.** Predictors of resilience profile membership

Reference group	Multidomain resilience				Low emotional behavioral resilience	
	Low cognitive resilience		Low emotional behavioral resilience		Low cognitive resilience	
	OR	95% CI	OR	95% CI	OR	95% CI
*Maltreatment characteristics*						
Abuse	.58	.28–1.20	.96	.59–1.57	.60	.28–1.30
Neglect	.79	.40–1.56	.90	.55–1.47	.88	.42–1.85
Out-of-home placement	.61	.27–1.41	1.70*	1.01–2.85	.36**	.16–.83
*Caregiver protective factors*						
Caregiver education (HS or more)	.28**	.12–.65	1.54	.81–2.94	.18***	.07–.47
Cognitive stimulation	.66***	.55–.80	.82**	.73–.92	.80*	.67–.97
Emotional support	.98	.82–1.17	.98	.89–1.07	1.00	.83–1.20
*Control variables*						
Child sex (girls)	.45*	.24–.85	.45**	.28–.72	1.00	.49–2.02
Child age	.05***	.02–.13	.68*	.49–.94	.08***	.03–.20
Child race/ethnicity^a^						
White/Non-Hispanic	1.16	.50–2.71	1.10	.64–1.90	1.06	.45–2.48
Hispanic	1.57	.61–4.03	1.26	.66–2.38	1.25	.45–3.47
Other^b^	.44	.06–3.04	1.23	.46–3.21	.36	.04–2.72
Household income (below FPL)	.83	.42–1.65	1.09	.67–1.78	.76	.37–1.59

*Note*. HS = high school; FPL = federal poverty level; OR = odds ratio.

a Black/Non-Hispanic is the reference group.

b Other race included American Indian, Asian, Native Hawaiian/Pacific Islander, and Multiple race categories.

* *p* < .05.

** *p* < .01.

*** *p* < .001.
